# Exposure to Cobalt Causes Transcriptomic and Proteomic Changes in Two Rat Liver Derived Cell Lines

**DOI:** 10.1371/journal.pone.0083751

**Published:** 2013-12-30

**Authors:** Matthew G. Permenter, William E. Dennis, Thomas E. Sutto, David A. Jackson, John A. Lewis, Jonathan D. Stallings

**Affiliations:** 1 Excet, Inc., Fort Detrick, Maryland, United States of America; 2 US Army Center for Environmental Health Research, Fort Detrick, Maryland, United States of America; 3 Naval Research Laboratory, Washington, District of Columbia, United States of America; University of South Florida College of Medicine, United States of America

## Abstract

Cobalt is a transition group metal present in trace amounts in the human diet, but in larger doses it can be acutely toxic or cause adverse health effects in chronic exposures. Its use in many industrial processes and alloys worldwide presents opportunities for occupational exposures, including military personnel. While the toxic effects of cobalt have been widely studied, the exact mechanisms of toxicity remain unclear. In order to further elucidate these mechanisms and identify potential biomarkers of exposure or effect, we exposed two rat liver-derived cell lines, H4-II-E-C3 and MH1C1, to two concentrations of cobalt chloride. We examined changes in gene expression using DNA microarrays in both cell lines and examined changes in cytoplasmic protein abundance in MH1C1 cells using mass spectrometry. We chose to closely examine differentially expressed genes and proteins changing in abundance in both cell lines in order to remove cell line specific effects. We identified enriched pathways, networks, and biological functions using commercial bioinformatic tools and manual annotation. Many of the genes, proteins, and pathways modulated by exposure to cobalt appear to be due to an induction of a hypoxic-like response and oxidative stress. Genes that may be differentially expressed due to a hypoxic-like response are involved in Hif-1α signaling, glycolysis, gluconeogenesis, and other energy metabolism related processes. Gene expression changes linked to oxidative stress are also known to be involved in the NRF2-mediated response, protein degradation, and glutathione production. Using microarray and mass spectrometry analysis, we were able to identify modulated genes and proteins, further elucidate the mechanisms of toxicity of cobalt, and identify biomarkers of exposure and effect *in vitro*, thus providing targets for focused *in vivo* studies.

## Introduction

Cobalt is a heavy metal with worldwide distribution that is found naturally in low concentrations and is used in many military and industrial applications. It is also a component of the essential vitamin B12, which is required for producing red blood cells. A cobalt deficiency, however, has not been described in humans [1], [2]. For most people, the largest source of cobalt is through the diet, ranging between 5 and 40 µg of cobalt per day [3]. An environmental exposure to higher levels may occur in particular industrial settings as cobalt is used as a pigment in glass, ceramics, and paints, and cobalt alloys are used in the production of aircraft engines, magnets, and artificial joints. Workers involved in metal mining, smelting, and refining may also be subject to higher levels of cobalt [4], [5].

A previous analysis by the Department of Defense assessed the potential oral (ingestive) hazards of industrial chemicals [6]. In this prioritization, an extensive database was created to access and prioritize the oral hazard of many different types of industrial chemicals. This assessment was based on the oral toxicity of a compound, its stability in the environment, its physical state, and its probability of being encountered based on industrial usage. A more detailed explanation of this prioritization is located in the referenced report. This prioritization considered many different types of chemicals, including pesticides and numerous organic and inorganic compounds. Originally, this database included data on 468 chemicals. However, for purposes of this paper, the database was limited to pure elements and metal compounds, which reduced the list to 36 entries, as shown in Table 1. As can be seen, based on its toxicity, its long-term stability in the environment, and its high probability score, cobalt dichloride is the highest scoring industrial chemical among all of the entries in this sub-class of industrial chemicals.

**Table 1:** Oral Toxicity Database.

Rank	Chemical	CAS Number	Toxic Hazard Score	Relative Probability Score	Total Score
1	Cobalt dichloride	7646-79-9	13	6	19
2	Magnesium (powder)	7439-95-4	9.5	9	18.5
3	Mercuric chloride	7487-94-7	15	3	18
4	Arsenic Trioxide	1327-53-3	14	4	18
5	Iodine	7553-56-2	13	5	18
6	Aluminum (powder)	7429-90-5	9.75	8	17.75
7	Red mercuric oxide	21908-53-2	13	4	17
8	Copper oxychloride	1332-40-7	12	5	17
9	Zinc	7440-66-6	10	7	17
10	Phosphorus	7723-14-0	12.75	4	16.75
11	Barium nitrate	10022-31-8	10.75	5	15.75
12	Cobalt (II) nitrate	10141-05-6	10.75	5	15.75
13	Potassium permanganate	7722-64-7	10.75	5	15.75
14	Silver nitrate	7761-88-8	9.75	6	15.75
15	Potassium nitrate	7757-79-1	8.75	7	15.75
16	Sodium Nitrate	7631-99-4	8.5	7	15.5
17	Thallium	7440-28-0	14	1	15
18	Thallium sulfate	7446-18-6	13	2	15
19	Arsenic	7440-38-2	12	3	15
20	Lead Oxide	1309-60-0	11	4	15
21	Mercuric nitrate	10045-94-0	11.75	3	14.75
22	Lead nitrate	10099-74-8	10.75	4	14.75
23	Mercury	7439-97-6	10.5	4	14.5
24	Sodium chlorate	7775-09-9	8.5	6	14.5
25	Cadmium	7440-43-9	10	4	14
26	Bismuth	7440-69-9	10	4	14
27	Aluminum phosphide	20859-73-8	9.5	3	12.5
28	Bromine	7726-95-6	8.5	4	12.5
29	Iron, pentacarbonyl	13463-40-6	9.25	2	11.25
30	Lead azide	13424-46-9	10	1	11
31	Arsenic trichloride	7784-34-1	9.25	1	10.25
32	Phosphotungstic acid	12067-99-1	6.25	3	9.25
33	Tetraethyl lead	78-00-2	8	1	9
34	Iodine pentafluoride	7783-66-6	7	2	9
35	Tetramethyl lead	75-74-1	5.75	1	6.75
36	Germane	7782-65-2	3.25	2	5.25

An extensive database was created to access and prioritize the oral hazard of industrial chemicals based on the toxicity, stability, and usage of the chemical. This prioritization was limited to only pure elements and metal compounds.

Cobalt is of particular interest to the military as it looks to replace depleted uranium with heavy metal tungsten alloys in ballistics due to environmental and public health concerns [7]. Heavy metal tungsten alloys contain 90 to 98% of tungsten by weight combined with nickel, iron, copper, and/or cobalt. The military was interested in these alloys due to their strength and supposed inertness [8]. However, in a study to investigate the health effects of these alloys, rats intramuscularly implanted with a weapons grade tungsten, nickel, and cobalt alloy developed aggressive metastatic tumors [9]. In a subsequent study, it was shown that these pellets are corrosive, unlike a tungsten, nickel, and iron alloy, increasing the availability of both nickel and cobalt [10]. Therefore, cobalt may play an important role in the carcinogenicity of this alloy.

Exposure to high concentrations of cobalt has been shown to cause adverse health effects in both animals and humans through various exposure routes. Cobalt can enter the body through respiration, ingestion, or contact with the skin. The adverse effects of an inhalation exposure occur mostly in the lung, however soluble cobalt ions can be released systemically [2]. An occupational exposure of metal workers caused respiratory effects in workers including respiratory irritation, diminished pulmonary function, asthma, and fibrosis. Although these workers were exposed to particles of metal alloys, the health effects were thought to be caused by cobalt ions which become more soluble when mixed with tungsten carbide [11]. Oral exposures can have systemic effects since cobalt is rapidly distributed into all tissues, and concentrated in the liver, kidney, and bone [12]. Children who were treated with oral cobalt doses of 1.4–10 mg cobalt/kg-day to treat anemia in the 1950’s developed depressed thyroid function and hyperplasia [13]. Animals treated with oral doses of cobalt were reported to have numerous effects, including cardiovascular, hematological, neurological, endocrine, and reproductive toxicity [14]. High serum cobalt ion concentrations are toxic to the liver, and increase aspartate aminotransferase (AST), alanine transaminase (ALT), and creatine kinase (CK) levels in mice [15]. Dermal exposures are unlikely to have systemic effects as cobalt cannot readily penetrate normal skin, although contact with cobalt can cause dermatitis [16].

While cobalt is known to cause adverse health effects, the exact mechanism of action remains unclear and biomarkers of exposure and effect have not been verified. A few potential mechanisms of cobalt toxicity include the induction of oxidative stress, induction of HIF-1α signaling, interference with ion homeostasis, and the disruption of glucose metabolism [17]–[21]. Changes in serum proteins have been identified as potential biomarkers of effect; however, the changes were not consistent, of large magnitude, or persistent [22]. Therefore, there is a need for further studies on the mechanisms of action of cobalt toxicity, as well as to identify biomarkers of effect to alert health care professionals to cobalt exposure prior to toxicological effects.

Therefore, we undertook a study to examine changes in gene expression and protein abundance to expand the understanding of the mechanisms of cobalt toxicity, as well as to identify potential biomarkers of exposure or effect. We exposed two well characterized rat liver derived cell lines, H4-II-E-C3 and MH1C1, to two concentrations of cobalt chloride and measured the gene expression changes using a DNA microarray and the changes in protein abundance in MH1C1 cells at one concentration of cobalt chloride using mass spectrometry. By focusing on the genes differentially expressed in both cell lines and combining the transcriptomic and proteomic data, we are able to describe modulated biological processes and genes and proteins involved therein. Additionally, extracellular proteins and genes which encode extracellular proteins were identified as potential candidate biomarkers of cobalt exposure or effect.

## Materials and Methods

### Cell Culture Conditions and Exposures

H4-II-E-C3 and MH1C1 cells (ATCC, Manassas, VA) were grown in Dulbecco’s Modified Eagle’s Medium (DMEM; Lonza, Walkersville, MD) supplemented with 10% fetal bovine serum (Invitrogen, Carlsbad, CA) and 2% Glutamax (Invitrogen) in a water-jacketed incubator at 37°C with 5% carbon dioxide. Confluent cultures in a T75 flask were exposed to CoCl_2_ (Sigma-Aldrich, St. Louis, MO). Exposure concentrations were chosen based on the CellTiter-Fluor Cell Viability assay (Figure S1, Promega, Madison, WI) and using a qPCR-based gene expression assay (Figure S2) [23]. Since we observed large gene changes at low or no levels of cytotoxicity, we used qPCR to aid in selecting definitive exposure concentrations. The final concentrations of CoCl_2_ were chosen to elicit a similar gene expression response as seen in previous studies [23], and were 160 and 310 µM for the MH1C1 cell line and 29 and 62 µM for the H4-II-E-C3 cell line. Different concentrations were used for each cell line due to differing results in both the viability and qPCR studies between the cell lines, which is a common occurrence [24]. Prior to exposure, cells were washed twice with serum- free DMEM to remove residual serum components. Fifteen milliliters of serum-free DMEM containing the proper concentration of CoCl_2_ were then added to each flask and cells were returned to the incubator for 24 h. Serum-free medium was used because conditioned media were also being collected for future secreted proteomic analysis, and the presence of large amounts of serum protein would interfere with this analysis [25]. Four biological replicates were performed for each condition, including the unexposed control.

### Microarray Preparation and Processing

Total RNA was extracted using Trizol solution (Invitrogen) applied directly to the flasks and an RNeasy Midi Kit cleanup (Qiagen, Germantown, MD) was performed to remove residual salts and organic solvents per the manufacturer’s instructions. RNA quality and quantity were determined using the Agilent Bioanalyzer Series II RNA 6000 Nano LabChip Kit and the 2100 Bioanalyzer (Agilent, Palo Alto, CA), and all samples were well within the criteria recommended by Affymetrix for use on a microarray. Using the Affymetrix 3′IVT kit, cDNA and labeled cRNA were prepared, washed, stained, and hybridized onto the Affymetrix Rat Genome 230 2.0 array and scanned per the manufacturer’s instructions.

### Mass Spectrometric Analysis of Intracellular Proteins

Separate MH1C1 exposures were completed for proteomic analysis. Cells were lysed using a hypotonic buffer as follows. Briefly, cells were rinsed with PBS and scraped from the surface of a T75 flask. The cell suspension was centrifuged at 450×g for 5 min, resuspended in hypotonic buffer (20 mM Tris, 5 mM MgCl_2_, 5 mM CaCl_2_, 1 mM DTT), and incubated for 30 min. The cell suspension was sonicated on ice using 20 second bursts 3 times at 20% power using a Vibra Cell (Sonics & Materials Inc., Danbury, CT), centrifuged for 20 min at 10,000×g, and the supernatant containing the intracellular proteins was collected. The proteins were digested with trypsin (Promega) using Rapigest (Waters, Milford, MA) to enhance the digestion, and salts and other contaminants were removed using Pierce Graphite Spin Columns per the manufacturer’s instructions (Thermo Scientific, Wilmington, DE). The protein mixture was quantified using a Nanodrop spectrophotometer at 280 nm (Thermo Scientific).

Seventy micrograms of protein was dried in a centrifugal evaporator, prepared for mass spectrometry according to the iTRAQ reagent protocol (SCIEX, Framingham, MA) and fractionated using a Polysulfomethyl A column (200 × 4.6 mm, 5 µm, 1000 Å, PolyLC, Columbia MD) at a flow rate of 0.95 mL/min. An Agilent 1100 HPLC equipped with an autosampler with an expanded injection loop and needle seat, diode array detector, fraction collector and Chemstation data system was used for the separation. The column compartment was set to 35°C and 1 mL of the sample was injected. The solvents used for the separation were solvent A (DI water), solvent B (500 mM ammonium formate, and 3% formic acid in water), and solvent C (acetonitrile). The initial conditions were 88%A: 2% B: 10% C. A linear gradient was performed to 90% B: 10% C in 20 min. The stop time was 30 min. One minute fractions were collected starting at 4 min and continuing until 28 min, producing 24 samples.

Separation of the peptides was performed on a Thermo Scientific Proxeon EASY nano-LC prior to analysis on a Thermo Orbitrap. The peptides were trapped using an Agilent Zorbax C-18 5×0.3mm, 5 µm particle size column. The analytical separation was performed using a Waters nanoAcquity UPLC column BEH C18 100 µm×100 mm, 1.7 µm particle. Ten microliters of the sample was loaded on the column and flushed with 60 µL of 0.1% formic acid in water. A gradient of 0.1% formic acid in water (Buffer A) and 0.1% formic acid in acetonitrile (Buffer B) was used. The gradient profile was as follows: initially 3% B, 45% B at 90 min, 95% B at 100 min, 95% B at 115 min, 3% B at 116 min, and the analysis stopped at 120 min. The flow rate was 0.30 µL/min. Precursor selection was done in a Thermo Orbitrap using 100,000 resolution. The top 10 peptides based on intensity were selected for fragmentation. Different energies, 40, 44 and 45 V for higher energy collision dissociation (HCD) were used in sequential runs of the same sample to increase the chances of reaching the most effective fragmentation energy for all proteins, allowing us to identify more peptides [26]. An exclusion list was created from the 40 V HCD and used to increase coverage on samples with more than 100 identified peptides on sequential runs. Species with single or unidentified charge states were excluded from precursor ion selection. Dynamic exclusion was used with a 20 sec window.

Mass spectral data was processed using Thermo Proteome Discoverer 1.3 (Thermo Scientific). Samples were searched using the Sequest generated randomized rat database based on all *Rattus norvegicus* sequences found in the National Center for Biotechnology Information Reference Sequence Database (4-12-2012), with the mass tolerance values set at 10 ppm for precursor ions and 0.8 Da for fragment ions [27]. Other parameters included fixed modifications on cysteine (methylthio), N-terminus (iTRAQ™ 8 plex), and lysine (iTRAQ™ 8 plex). Variable modifications were applied to methionine (oxidation), serine/tyrosine/threonine (phosphorylation) and tyrosine (iTRAQ™ 8 plex). Scans having peptide identifications with a 1% false discovery rate (FDR) were used for quantification. Reporter ions were quantified with a mass tolerance window of 20 ppm using the most confident centroid. Reporter ion intensities were corrected using the Discoverer 1.3 default method of iTRAQ™ 8-plex mass tags by Applied Biosystems optimized for Thermo Scientific.

### Data Analysis

Microarray data was processed for background adjustment, normalization, and summarization by the Robust Multi-Array Averaging method (RMA) [28] using Partek Genomic Suite (GS) software (Version 6.6, St. Louis, MO). All data is compliant with the Minimum Information About a Microarray Experiment (MIAME) guidelines, and the raw data files can be found in the NCBI Gene Expression Omnibus (accession number GSE51207). The microarray data was examined for outliers using a principal component analysis (PCA) in Partek GS. Pairwise correlation analysis between replicates and inter-replicate dot plots of all probe sets were performed to verify reproducibility. Replicates with an R^2^>0.95 and no gross deviations from linearity on the dot plot were accepted. A present, absent, or marginal detection call for each probe set was determined using the Affymetrix GCOS algorithm, and only probe sets with a present detection call for all samples in at least one condition were retained for analysis [29].

For each cell line, an analysis of variance (ANOVA) was performed with the dose as the variable using the contrast function in Partek GS between each condition and control to determine which genes were differentially expressed due to treatment. Probe sets were retained for bioinformatic analysis with a Benjamini and Hochberg FDR ≤0.01 for the concentration variable and a 1.8 or greater fold change from control in at least one treatment condition [30].

Scaffold Q+ (version Scaffold_4.0.4, Proteome Software Inc., Portland, OR) was used to quantitate the iTRAQ labeled peptide and protein identifications. Peptide identifications with a greater than 92.0% probability to achieve an FDR<1.0% by the Scaffold Local FDR algorithm were accepted. Protein identifications were accepted if they could be established at greater than 99.0% probability and contained at least two identified peptides. Protein probabilities were assigned by the Protein Prophet algorithm [31]. Proteins that contained similar peptides and could not be differentiated based on MS/MS analysis alone were grouped to satisfy the principles of parsimony. Proteins sharing significant peptide evidence were grouped into clusters. Channels were corrected by the default SCIEX purity corrections in all samples according to the algorithm described in i-Tracker [32]. Acquired intensities in the experiment were globally normalized across all acquisition runs. Individual quantitative samples were normalized within each acquisition run. Intensities for each peptide identification were normalized within the assigned protein. The reference channels were normalized to produce a 1∶1 fold change. All normalization calculations were performed using medians to multiplicatively normalize data. Differentially expressed proteins were determined using Permutation Test analysis using Bonferroni multiple testing correction.

Ingenuity Pathway Analysis (IPA) software (Ingenuity Systems, www.ingenuity.com) was used to explore the biological implications of the data. Core analyses were performed using all present probe sets as the reference set for transcriptomic data, all identified proteins as the reference set for the proteomic data, and all present probe sets and identified proteins mapped by IPA as the reference set for the combined data, with all other default settings selected. We considered IPA canonical pathways, functions, and other enrichments statistically significant with a p-value ≤0.05 and involving more than two molecules, and transcription factors were considered significant with an activation z-score <−2 or >2 and a p-value of overlap ≤0.05. Differentially expressed genes were also examined manually on an individual basis.

## Results and Discussion

Since cobalt is an environmental and military occupational hazard [6], we undertook this study to further elucidate the molecular mechanisms of action and identify potential biomarkers of exposure and effect. We performed a global analysis of transcripts and protein abundance and investigated the molecular pathways altered in two rat hepatoma derived cell lines (H4-II-E-C3 and MH1C1) exposed to CoCl_2_ for 24 h. Integrated transcriptomic and proteomic analysis identified four major biological effects: response to oxidative stress, response to damaged proteins, energy metabolism, and Hif-1α signaling. We propose candidate biomarkers for future validation.

### Microarray and Mass Spectrometry Analysis

**Figure 1:**
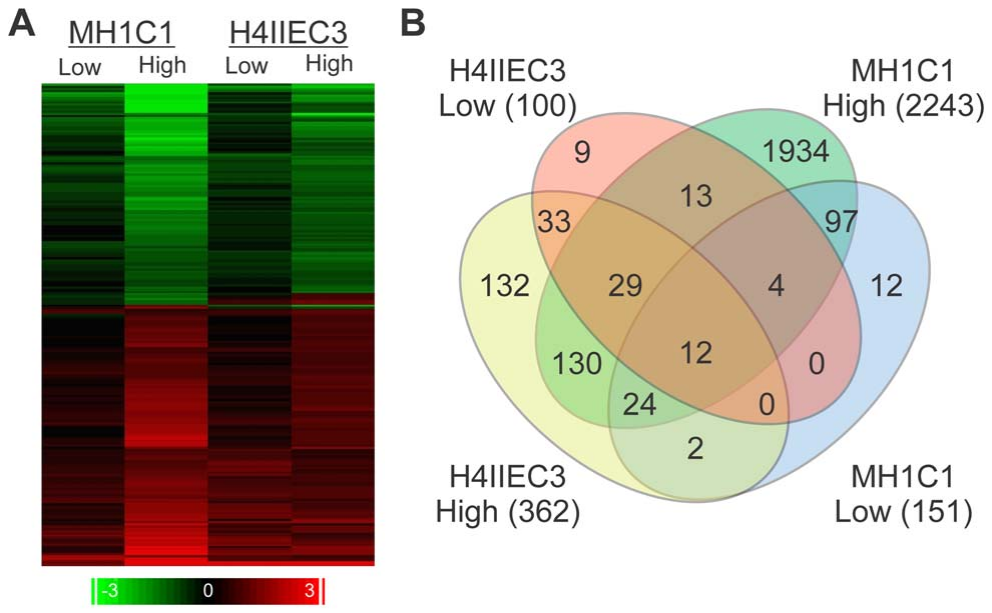
Differentially expressed probe sets. A total of 2,431 probe sets are differentially expressed at a FDR<0.01 and a 1.8 fold change cut off in at least one condition. The 212 probe sets that are differentially expressed in both cell lines are shown in the heat map with the log_2_ ratio of change displayed.

To identify genes differentially expressed due to exposure to cobalt, we measured mRNA using whole-genome, DNA oligonucleotide microarrays. For each cell line, the data were preprocessed using the RMA method and filtered to select only probe sets with a present call in all replicates of at least one condition, which retained 17,563 probe sets for MH1C1 cells and 16,992 for H4-II-E-C3 cells out of the 31,099 possible probe sets on the microarray for further analysis. We selected differentially expressed probe sets using an ANOVA with a Benjamini-Hochberg FDR ≤0.01 and a fold change filter of ≥1.8 for each cell line independently. In the MH1C1 cell line we identified 151 and 2,243 probe sets in the low and high concentration, respectively. In the H4-II-E-C3 cell line we identified 100 and 362 probe sets in the low and high concentration, respectively (Table S1). In order to remove cell type specific effects and identify changes caused by chemical insult in a hepatocyte-like background, we focused on the 214 probe sets that are differentially expressed in at least one condition of both cells lines (Figure 1). Of the 214 differentially expressed probe sets, 114 were up-regulated, 93 were down-regulated, and 7 were regulated in conflicting directions between the two cell lines.

Using mass spectrometry, we identified intracellular proteins changing in abundance after exposure to cobalt chloride in MH1C1 cells. We examined the high concentration of the MH1C1 cells since this dose and cell line had the greatest effect with regard to gene expression changes, which allowed us to identify a large number of proteins changing in abundance. We identified a total of 1680 proteins with 99.0% probability that contained at least two identified peptides. Using the Permutation Test analysis with a Bonferroni multiple testing correction p-value<0.05 and a fold changed cutoff of 1.5 in at least one sample, we identified 56 proteins that changed in abundance (Table S2), of which 43 increased and 13 proteins decreased. We also examined the overlap of the proteomic and transcriptomic data, finding 10 matches between the two data sets. The gene names of the 10 matching molecules are: annexin A2; glutathione S-transferase A2; heat shock 70kD protein 1A; heme oxygenase (decycling) 1; myosin light chain kinase; phosphofructokinase, liver; phosphoglycerate kinase 1; proteasome (prosome, macropain) subunit, beta type 3; solute carrier family 2 (facilitated glucose transporter), member 1; and ubiquitin-conjugating enzyme E2H. The intersection of the transcriptomic and proteomic data is under-represented due to the strict criteria we used to determine whether or not a gene is differentially expressed. Therefore, we compared only fold change values, and found that 31 of the 56 changing proteins have corresponding transcripts that are changing by at least 1.5 fold in the same direction, providing a stronger agreement between the two approaches.

### Bioinformatic Analysis

We used IPA to further explore the biological meaning of the differentially expressed genes and proteins. When examining only the transcriptomic data, 15 IPA canonical pathways are significantly enriched, and two transcription factors are predicted to be activated. When proteomic data alone was analyzed, four IPA canonical pathways are enriched and three transcription factors are predicted to be activated. As the transcriptomic and proteomic data do not completely overlap, we performed an analysis by combining the two datasets, resulting in 14 enriched IPA canonical pathways and one inhibited and four activated transcription factors (Table 2 and S3).

**Table 2:** Enriched Pathways.

Transcriptomic		Proteomic		Combined	
IPA Canonical Pathway	−log pvalue	IPA Canonical Pathway	−log pvalue	IPA Canonical Pathway	−log pvalue
Acute Phase Response Signaling	4.25	Glycolysis I	5.69	Glycolysis I	7.22
LXR/RXR Activation	3.97	Gluconeogenesis I	4.54	Gluconeogenesis I	5.96
NRF2-mediated OxidativeStress Response	3.24	NRF2-mediated Oxidative Stress Response	2.22	LXR/RXR Activation	4.27
Maturity Onset Diabetes ofYoung (MODY) Signaling	2.81	Aryl Hydrocarbon Receptor Signaling	1.67	Maturity Onset Diabetes ofYoung (MODY) Signaling	4.00
Antioxidant Action of Vitamin C	2.79			NRF2-mediated OxidativeStress Response	3.90
Glycolysis I	2.75			Acute Phase Response Signaling	3.53
PXR/RXR Activation	2.44			Antioxidant Action of Vitamin C	2.28
Gαq Signaling	2.41			TR/RXR Activation	2.21
TR/RXR Activation	1.98			AMPK Signaling	2.05
AMPK Signaling	1.89			Complement System	2.03
Xenobiotic Metabolism Signaling	1.82			Gαq Signaling	1.92
Aldosterone Signaling in Epithelial Cells	1.72			PXR/RXR Activation	1.90
HIF1α Signaling	1.67			14-3-3-mediated Signaling	1.47
Renal Cell Carcinoma Signaling	1.50			IL-10 Signaling	1.35
Protein Ubiquitination Pathway	1.42				

Enriched IPA canonical pathways are listed for the transcriptomic, proteomic, and combined data. We considered a pathway to be enriched at a p<.05 and contain more than 2 changing molecules.

**Figure 2:**
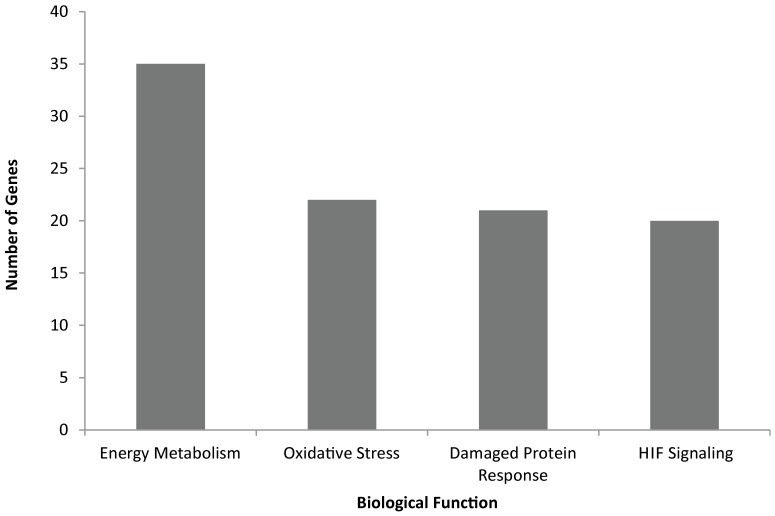
Overarching biological processes affected by Cobalt exposure. In order to assign function to more differentially expressed genes, we created four biological functions consistent with the enrichment analysis and literature on cobalt toxicity. The four categories are energy metabolism, oxidative stress, unfolded protein response, and HIF Signaling. A gene may be present in more than one category.

Upon initial review of these findings, it was evident that conventional gene ontology and pathway categories failed to completely describe the biological responses since only a small number of genes were present in each pathway or category and many of the genes were present in more than one. Therefore, we manually annotated the genes using the available literature, and found that many genes are related to four overarching processes: the response to oxidative stress, the response to damaged proteins, energy metabolism, and HIF-1α signaling, consistent with known effects of cobalt toxicity (Figure 2) [17]–[21], [33].

### Hif-1α Signaling and Energy Metabolism

**Figure 3:**
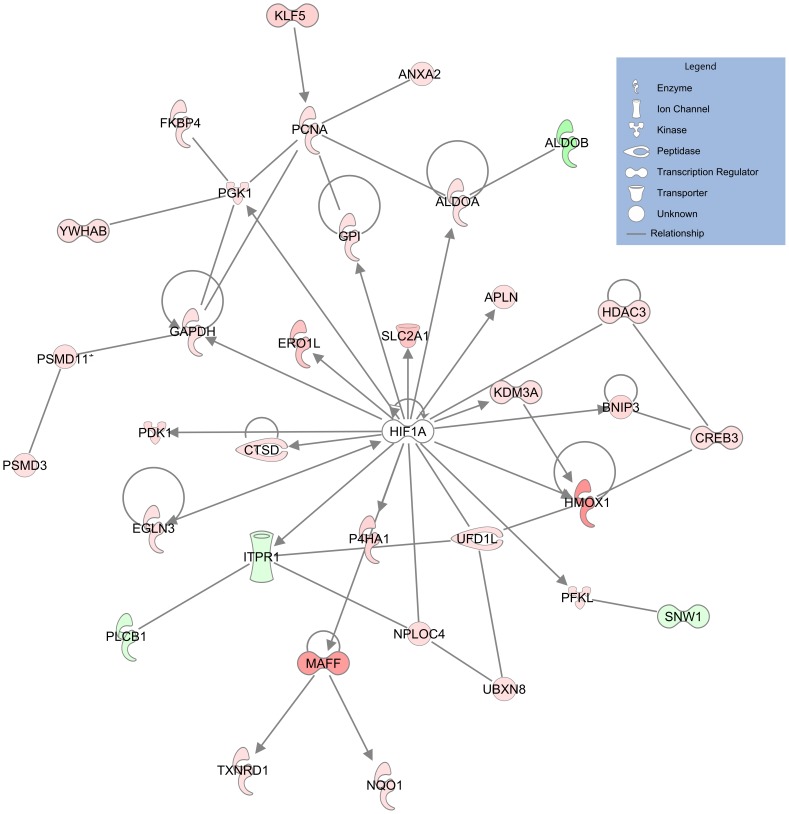
Network of modulated genes and proteins related to HIF-1α. Many of the differentially expressed genes and/or proteins changing in abundance are regulated by HIF-1α. The arrows show the direction of the relationship, the color indicates the direction of change of the gene or protein with red being an increase and green being a decrease, and the intensity of the color indicating the degree of change.

The observed gene expression and protein changes are consistent with the known ability of cobalt to induce a hypoxic-like response [21]. In our data, the HIF-1α signaling IPA canonical pathway is enriched at the transcript level (p = 0.02). Furthermore, in the IPA transcription factor analysis HIF-1α is predicted to be activated by the perturbed genes, proteins, and combined data (z-score = 2.6, 2.6, and 3.1 respectively). Manual annotation also identified many genes known to be regulated by HIF-1α (Figure 3), and therefore may be differentially expressed as a result of HIF-1α stabilization, and accounting for almost ten percent of the differentially expressed genes.

Cobalt chloride is thought to induce a hypoxic response by stabilizing HIF-1α through disruption of the hydroxylases responsible for HIF-1α degradation by replacing the iron in the active site of the hydroxylases or by depleting ascorbate, which oxidizes the iron and inactivates the enzyme [34], [35]. In particular, egl nine homolog 3 (Prolyl hydroxylase 3; PHD3 or EGLN3) regulates HIF-1α degradation, and is also transcriptionally up-regulated by HIF-1 α, perhaps by forming a feedback loop [36]. Indeed, del Peso and colleagues reported that HIF-1α is essential for the up-regulation of EGLN3, as it is not induced in cells lacking a functional HIF-1α [36]. *Egln3* is up-regulated in our microarray data, which is consistent with this feedback loop model. Therefore, we provide further evidence that cobalt activates HIF-1α.

HIF-1α activation is also known to effect cellular energy metabolism as the cell shifts toward non-oxidative forms of ATP production through the regulation of glycolytic enzymes and glucose transporters [37]. Our data supports a shift of the cells towards glycolysis as would occur during a hypoxic-like response. For the proteomic data and the combined transcriptional and proteomic data, Glycolysis I is the most significantly enriched IPA canonical pathway (p<0.001), and is also enriched for the transcriptional data alone (p = 0.002). Many critical genes in the glycolytic pathway are differentially expressed, including glycogen synthase (*Gys1*), phosphoglucomutase 1 (*Pgm1)*, phosphoglycerate kinase 1 (*Pgk1)*, and pyruvate dehydrogenase kinase, isozyme 1(*Pdk1)*. Furthermore, the gene expression and protein abundance of the key enzyme in the glycolysis pathway, phosphofructokinase, liver (PFKL) is up-regulated. PFKL catalyzes the transfer a phosphate group from ATP to fructose-6-phosphate forming fructose-1,6-bisphosphate. As this step is irreversible and rate limiting, it is considered a key control point of glycolysis, ensuring the reverse process, gluconeogenesis, does not occur [38]. Therefore, we provide further insight into the ability of cobalt chloride to regulate energy metabolism, perhaps due to the stabilization of HIF-1α, by increasing glycolysis through increased expression of key genes in the pathway.

Our data is consistent with the ability of cobalt to interfere with hypoxic signaling pathways. We identify HIF-1α regulated genes, pathways, and proteins, as well as transcriptional changes in genes encoding key enzymes that regulate the energy metabolism of the cell due to a hypoxia-like response.

### Oxidative Stress

The enriched pathways and perturbed genes and proteins in our work support an induction of oxidative stress by cobalt and provides information on the cellular response to such stress. Cobalt has previously been shown to induce oxidative stress through generation of reactive oxygen species in cells by Fenton-like reactions [39]. The IPA canonical pathway NRF2-mediated oxidative stress response is a significantly enriched pathway in the transcriptomic and proteomic data alone and when combined (p<.001, 0.006, and <0.001, respectively). Additionally, the NRF2 transcription factor is predicted to be activated in the IPA transcription factor analysis for all data sets (z-score = 3.3, 2.57, and 3.8 for the transcriptomic, proteomic, and combined data, respectively). Therefore, our data support NRF2 as a key regulator of the response to cobalt induced oxidative stress. In normal conditions, NRF2 is retained in the cytoplasm and targeted for proteasomal degradation by interaction with its suppressor kelch-like ECH-associated protein 1 (KEAP1) [40]. This interaction is abolished by oxidative stress, stabilizing NRF2, which then accumulates in the nucleus [40]. NRF2 downstream targets are thought to protect against oxidative stress through three main methods: the detoxification of oxidants and xenobiotics, the reduction of oxidized proteins, or the production of antioxidants [41]. Our data suggests the NRF2 pathway is indeed activated in response to oxidative stress with downstream target genes for at least two of the three detoxification methods.

Our data describes differentially expressed genes and proteins as well as enriched IPA pathways involved in the NRF2 mediated detoxification and metabolism of oxidants, such as NADPH dehydrogenase, quinone 1(*Nqo1*), glutathione S-transferase, and superoxide dismutase (*Sod1*). NQO1 catalyzes the two-electron reduction of quinones, thereby depressing the levels of quinones and preventing their one electron reduction, which would result in the production of radical species [42]. Glutathione S-transferase is involved in the detoxification of electrophiles through the conjugation of such compounds with glutathione [43]. SOD1 converts the toxic superoxide radicals to less toxic molecular oxygen and hydrogen peroxide [44]. The perturbation of these genes and proteins, as well as the IPA predicted activation of NRF2, support the NRF2 mediated detoxification of oxidants induced by exposure to cobalt.

Perturbed genes and proteins in our work may also produce antioxidants as a result of the NRF2 mediated response. As the transcript for glutathione S-transferase is upregulated to form glutathione conjugated compounds, so is the transcript and protein glutamate-cysteine ligase (*Gclm)*, which catalyzes the first rate limiting step in glutathione synthesis [45]. Furthermore, heme oxygenase, which has the greatest fold change in both the transcriptomic and proteomic data, is known to be regulated by NRF2 and involved in the production of the antioxidant bilirubin [46]. Therefore, our data suggests that exposure to cobalt induces the expression of genes and increases the abundance of proteins involved in the NRF2 mediated production of antioxidants to attenuate oxidative stress.

We also identify transcriptional and protein abundance changes that indicate cellular proteins are damaged by cobalt induced oxidative stress. Mammalian cells have only a limited protein repair mechanism; consequently most damaged proteins undergo degradation. The proteasome is often responsible for the degradation of damaged proteins, and does so by recognizing hydrophobic amino acid residues that are exposed by oxidative damage and cleaving them in an ATP and ubiquitin-independent pathway, therefore playing a central role in the cell’s antioxidant defense [47]. Many genes encoding sub units of the proteasome are up-regulated in response to cobalt in this study, including *Psmb3*, *Psmd11*, *Psmb2*, and *Psmd3*. Hence, increased proteasomal degradation may be a result of protein damage by cobalt induced reactive oxygen species.

Cobalt is known to produce oxidative stress through the production of reactive oxygen species; undergoing a Fenton-like reaction. We are able to identify transcripts and proteins changing in abundance likely in response to this stress indicating the activation of the NRF2 transcription factor and induction of the downstream targets of Nrf2, as well as an increase in the proteasomal degradation pathway to rid the cell of damaged proteins.

The cobalt induced oxidative stress and protein damage may also disrupt the proteostasis network which regulates the folding of newly synthesized proteins, remodeling of misfolded proteins, and the degradation of damaged proteins. Environmental stress, such as an exposure to metals, can cause an accumulation of misfolded or oxidized proteins. The cells may form an adaptive response to eliminate the damaged proteins or the stress may overwhelm the ability of the cell to restore normal proteostasis, leading to a dysfunctional state [48], [49]. Our data does not indicate whether the exposed cells have formed an adaptive response or are in a dysfunctional state, or if they would be able to return to a normal state upon removal of the toxicant.

### Zinc and Iron Substitution

Many of the toxic effects of cobalt may be due to cobalt substituting for zinc or iron in proteins, thus disrupting their normal function. In general, metal ions have been shown to disrupt the function of zinc finger proteins through displacement, the formation of mixed complexes, or through the oxidation of the metal binding domain [50]. Particularly in this work, the stabilization of both HIF-1α and NRF2 may be due to this substitution. Cobalt has been shown to replace iron in the hydroxylase responsible for the degradation of HIF-1α, which then accumulates in the cell and activates downstream targets [34]. Similarly, NRF2 is sequestered in the cytoplasm and targeted for degradation by KEAP1, a zinc metalloprotein [40], [51], in which cobalt has been shown to substitute for zinc [52]. While it remains unclear if the disruption of the HIF-1α and NRF2 inhibitors is due to the substitution of cobalt for iron or zinc, the depletion of ascorbate, damage by oxidative stress, or another method, it is also possible that many of the toxic effects of cobalt are due to the inactivation of metalloproteins, which is an area in need of further investigation.

### Biomarkers

One of the goals of this work is to identify potential biomarkers of exposure and effect. Extracellular proteins make attractive candidates for biomarkers since a biomarker is ideally easily accessible. Intracellular liver proteins would most likely only be accessible through liver biopsy unless released as leakage products, while liver-secreted extracellular proteins are more likely to be detectable in the blood, as the organ is a highly vascularized. Therefore, we identified genes and/or proteins changing in abundance due to exposure to cobalt that are predicted to be present in the extracellular space and may be useful as candidate biomarkers of exposure or effect (Table 3).

**Table 3:** Modulated Extracellular Transcripts and Proteins.

Gene Symbol	Gene Name
APLN	apelin
APOB	apolipoprotein B
AZGP1	alpha-2-glycoprotein 1, zinc-binding
BTD	biotinidase
C4BPA	complement component 4 binding protein, alpha
C5	complement component 5
C8A	complement component 8, alpha polypeptide
Ces1c	carboxylesterase 1C
CP	ceruloplasmin (ferroxidase)
CXCL9	chemokine (C-X-C motif) ligand 9
FBXO30	F-box protein 30
FGA	fibrinogen alpha chain
GPI	glucose-6-phosphate isomerase
ITIH1	inter-alpha-trypsin inhibitor heavy chain 1
ITIH3	inter-alpha-trypsin inhibitor heavy chain 3
ITIH4	inter alpha-trypsin inhibitor, heavy chain 4
Mug1/Mug2	murinoglobulin 1/2
NAMPT	nicotinamide phosphoribosyltransferase
Pzp	pregnancy zone protein
SERPINA1	serine (or cysteine) proteinase inhibitor, clade A (alpha-1 antiproteinase, antitrypsin), member 1
SERPINA11	serine (or cysteine) peptidase inhibitor, clade A (alpha-1 antiproteinase, antitrypsin), member 11
SERPINA6	serine (or cysteine) peptidase inhibitor, clade A, member 6
SLC39A10	solute carrier family 39 (zinc transporter), member 10
SPP2	secreted phosphoprotein 2
TTR	transthyretin
VTN	vitronectin

We identified 26 extracellular proteins and/or genes which encode extracellular protein whose expression was modulated in response to cobalt exposure. We propose them as candidate biomarkers of exposure or effect. We focused on extracellular proteins as they have the best potential to be identified through non-invasive methods.

A biomarker also needs to have a detectable change in order for it to be useful as a diagnostic tool. Of the 26 potential biomarker candidates we identified, the five with the greatest fold change were identified as changing transcripts and are complement component 5 (*C5*); solute carrier family 39 (zinc transporter), member 10 (*Slc39a10*); fibrinogen alpha chain (*Fga*); serpin peptidase inhibitor, clade A, member 1 (*Serpina1*); and apolipoprotein B (*Apob*). C5 is involved in the complement cascade, and has also been shown to play a role in chronic toxic liver injury [53], but interestingly it is down-regulated almost 13-fold at its maximum in our study. SLC39A10 is a zinc transporter that has been found in plasma [54], and may be down- regulated to control the ion homeostasis of the cell [55] which has been altered due to cobalt induced oxidative stress. FGA is a component of fibrinogen, a major fraction of blood clots, has been identified as differentially expressed during liver fibrosis [56], suggesting that FGA could be a biomarker of cobalt induced liver injury. SERPINA1 is an abundant plasma protein synthesized in the liver associated with inflammation, trauma, and pregnancy [57]. Interestingly, it has also been reported to be decreased with increasing oxidative stress at the protein level due to fragmentation, misfolding, polymerization, and aggregation [58], and is also down- regulated in our transcript data. APOB catalyzes the rate-limiting step in hepatic very low density lipoprotein formation. Recently, APOB has also been shown to be decreased in response to ER stress and the unfolded protein response [59], and may therefore serve as a biomarker for cobalt toxicity in response to unfolded or damaged proteins or the misregulation of energy metabolism. We suggest the secreted proteins encoded by these genes are worthwhile candidates to be pursued as biomarkers of cobalt exposure and effect.

### Caveats of Analysis

Using enrichment analysis tools such as IPA allows us to identify perturbed pathways, biological functions, and transcription factors of cobalt exposure; however, there are many limitations when using this tool. Perhaps our biggest concern when using IPA and other enrichment tools is that they only describe a small percentage of data, a limitation inherent to most pathway tools. We identified 138 unique, annotated, differentially expressed genes and 58 proteins changing in abundance, yet the IPA canonical pathway analysis only describes the function of 43 genes and/or proteins. However, using manual annotation we were able to assign an overall function to most of the differentially expressed genes. Additionally, we can assign the four major biological functions discussed in this work (response to damaged proteins, Hif-1α signaling, energy metabolism, and oxidative stress response) to about 60% of our differentially expressed genes, further confirming our findings.

In this work we examined the intracellular proteins that change due to exposure to cobalt. The purpose of our study is to further elucidate the mechanism of toxicity of exposure to cobalt; identify the perturbed pathway, genes, and proteins; and identify potential biomarkers of exposure or effect for further study. While examining intracellular proteins provides insight into the modulated cellular processes and functions, it may not be the optimal method for biomarker discovery, as it does not measure the secreted proteins. In our work, only one of the proteins changing in abundance is known to be a secreted protein and the remaining are all intracellular. Although it too has limitations, a more efficient method of biomarker discovery may be to examine proteins in the conditioned media of the cell exposures. However, examining conditioned media also has limitations and challenges, and may not be the best method to investigate the molecular methods of toxicity.

Another caveat to our work is that we conducted all experiments using serum free growth medium, and the presence of serum in the medium would most likely reduce the bioavailability of the metal. We did not model the concentrations to *in vivo* exposures where the presence of serum would be a factor, but instead chose concentrations of cobalt based on a measurable biological response. It is possible that these *in vitro* concentrations are irrelevant to *in vivo* exposures, however experiments are currently underway in our lab to determine parameters that will allow future *in vitro* work to be modeled based on *in vivo* exposure evidence.

## Conclusion

We exposed two rat liver derived cell lines to cobalt chloride and interrogated gene expression and alterations in intracellular protein abundance to elucidate mechanisms of toxicity and identify candidate biomarkers. Performing enrichment analysis allowed us to identify perturbed canonical pathways and potential transcription factor activation or inhibition, and identify differentially expressed genes which may serve as potential biomarkers.

Using enrichment analysis we identified four main components of cobalt toxicity; a response to damaged proteins, HIF-1α signaling, energy metabolism, and oxidative stress response. We identified differentially expressed genes that may be directly regulated through HIF-1α, as well as many genes involved in energy metabolism that may be changing in response to a hypoxic-like environment. Furthermore, many of the differentially expressed genes in our data may be directly involved with minimizing the damage of cobalt induced oxidative stress or in the degradation of proteins that are damaged. We also identified novel candidate biomarkers that upon validation could prove useful in exposure assessment, as well as to identify therapeutic points of intervention. This work identifies key genes, transcription factors, and pathways involved in the cells’ response to cobalt intoxification.

## Supporting Information

Figure S1 **Cell viability assay results for rangefinding.** Cell viability rangefinding studies were conducted to ensure that concentrations used for gene expression analysis were at sub-lethal concentrations. The points not connected to a line denote the low and high concentrations used in the definitive studies.
(TIF)

Figure S2 **qPCR results used for rangefinding.** We determined the gene expression for a panel of genes using semi-quantitative qPCR to aid in selecting doses for the definitive experiment. The points not connected to a line denote the low and high concentrations used in the definitive studies.
(TIF)

Table S1 Differentially expressed probe sets. We considered a probe set to be differentially expressed with a FDR≤0.01 and a fold change of at least 1.8 from control. Each worksheet lists the differentially expressed genes for each cell line at each concentration, with the last worksheet listing the 214 common differentially expressed probe sets.
(XLSX)

Table S2 Proteins changing in abundance. We identified 56 proteins that changed in abundance, of which 43 increased and 13 proteins decreased, with a p-value<0.05 and a fold changed cutoff of 1.5 in at least one sample.
(XLSX)

Table S3 Significant transcription factors. IPA transcription factors that are predicted to be activated or inhibited are listed on separate worksheets for the transcriptomic, proteomic, and combined data. We considered an activation z-score >2 or <−2 to be significant.
(XLSX)
